# Reversal for metabolic syndrome criteria following the CHANGE program: What are the driving forces? Results from an intervention community-based study

**DOI:** 10.3934/publichealth.2025011

**Published:** 2025-01-21

**Authors:** Hanan E. Badr, Travis Saunders, Omar Bayoumy, Angelie Carter, Laura Reyes Castillo, Marilyn Barrett

**Affiliations:** 1 Department of Applied Human Sciences, Faculty of Science, University of Prince Edward Island, Charlottetown, Canada; 2 Health and Wellness Centre, University of Prince Edward Island, Charlottetown, Canada

**Keywords:** metabolic syndrome, hypertension, HbA1c, triglycerides, HDL, waist circumference, abdominal obesity, diabetes mellitus

## Abstract

**Objective:**

To examine the impact of the Canadian Health Advanced by Nutrition and Graded Exercise (CHANGE) program on the reversal of one or more metabolic syndrome (MetS) criteria among community members with MetS and define the significant predictors of upholding individual MetS criterion from 2020 to 2023.

**Methods:**

The program enrolled 278 community members with/or at risk of MetS. Participants followed regular physical activity and a Mediterranean diet for 12 months with the assistance of a registered dietitian and a kinesiologist. A licensed practical nurse and/or registered nurse measured participants' weight, height, waist circumference, and blood pressure and withdrew blood samples for laboratory investigations. Physical activity, physical fitness, Mediterranean diet score (MDS), anthropometric measurements, and laboratory investigations were assessed at the baseline and every three months. Descriptive statistics were calculated, and binary logistic regression analysis was performed to define the significant predictors of upholding each criterion of the MetS.

**Results:**

Participants' mean age was 60.5 ± 11.7 years, and 74.8% were females. Participants with MetS decreased by 5.04% by the end of the program. The percentage of participants with each MetS criterion showed a significant decrease at the end of the study, except for low HDL, which remained with no change. Moreover, the mean of physical activity, physical fitness tests, and MDS scores showed a significant increase after the 12 months of study. Compared to baseline, daily sedentary and screen times showed a significant decrease at the end of the program (8.6 *vs*. 7.2 and 3.2 *vs*. 2.6 hours, respectively). Logistic regression analysis revealed that age, female gender, low educational attainment, physical activity, physical fitness, and screen time were significant predictors for upholding one or more MetS criteria.

**Conclusion:**

MetS criteria can be reversed following the CHANGE program. Sociodemographic and lifestyle features are significant predictors for upholding MetS criteria. The program is cost-effective considering its low cost and could lead to significant savings on healthcare costs. Further studies among different communities are recommended to confirm the generalizability of the results.

## 1. Introduction

Approximately one in five Canadian adults has metabolic syndrome (MetS) [1], a cluster of risk factors for diabetes and heart disease, which significantly increases their risk for cardiovascular disease, diabetes, cancer, and all-cause mortality [2],[3]. To be diagnosed with metabolic syndrome, an individual must demonstrate at least three of the following risk factors: elevated blood pressure, blood glucose and triglycerides and/or receiving medication for any of them, abdominal obesity, and low HDL cholesterol [4],[5]. Previous studies have consistently demonstrated that lifestyle modifications, including diet and/or exercise, can reduce the prevalence of individual risk factors of the MetS itself [4],[6],[7]. However, few previous studies report the impact of programs run in primary care settings, instead focusing primarily on controlled research studies [6],[7]. Further, these studies have typically been run in large centers with populations well above >100,000 individuals. While this type of design may provide high-quality data, it also limits the generalizability of the findings to primary care settings, especially for more rural centers. In addition, there is a gap in knowledge regarding which components of the lifestyle amendment programs have an impact on improving MetS criteria. In addition, previous studies illustrated the role of sociodemographic factors, such as age and gender, in the prevalence of MetS [8],[9]. However, other personal characteristics are less examined. The current study will help in filling these knowledge gaps.

Based in Charlottetown, Prince Edward Island (PEI) (population: 36,000), the University of PEI Health and Wellness Centre provides primary care services to all students, faculty, and staff affiliated with the institution. Since 2020, the Health and Wellness Centre has also offered the CHANGE program, a 1-year diet and exercise intervention to community members with two or more metabolic risk factors [10]. The program is delivered by a kinesiologist and registered dietitian (RD) and offered for free at 7–8 sites across PEI. With only one city exceeding a population of 30,000 and a provincial population of just 174,000 island-wide [11], PEI is largely rural, and by far Canada's smallest province. Further, just 76% of Islanders have a family physician, which represents the lowest access of any Canadian province [12]. The CHANGE intervention provides an ideal opportunity to examine the impact of a primary care-based lifestyle intervention for individuals with metabolic syndrome living in a rural area with limited access to healthcare services. Because the program was primarily designed as a community program (and not as a research project), the results are directly relevant to other primary care settings. The purpose of this analysis was therefore to examine the impact of the CHANGE program on the reversal of one or more metabolic syndrome criteria among community members and to define the significant predictors of upholding individual metabolic syndrome criteria from 2020 to 2023.

## 2. Materials and methods

### 2.1. Study design and study population

This prospective study was conducted in Prince Edward Island, Canada, and encompassed three cohorts of the CHANGE program, a community-focused exercise and diet program provided by the Health and Wellness Centre at the University of Prince Edward Island. Over the three years, 575 community members were screened for MetS criteria, with an overall sample of 363 eligible participants (63.1% of the screened members). The sample size was calculated using proportional allocation to be mostly representative of the PEI population to avoid potential sampling bias as much as possible. A total of 278 individuals completed the CHANGE 12-month program and were included in the current analysis, while 85 participants dropped out due to different reasons such as conflict with working times, travel, or developed health issues/injuries discrete from the program. The program was funded by a combination of government funding and private donations, with a total cost of $474,000 for the first three cohorts ($158,000/cohort).

An adult participant was eligible to be enrolled in the CHANGE program if aged at least 18 years, spoke and read English clearly, and screened positive for at least two of the five MetS criteria according to the Canadian Guidelines of MetS [5]. Although the guidelines recommend that a person should have at least three out of five MetS criteria to be considered to have MetS, the program enrolled 34 people (12.2%) who met only two criteria considering them at risk for MetS to protect them from developing MetS. On the other hand, pregnant women and people with Type 1 diabetes mellitus were excluded from the study.

### 2.2. The CHANGE program

This one-year supervised diet and exercise program was developed by MetS Canada targeting people with MetS. The program was adapted to the PEI setting and conducted by the University of Prince Edward Island (UPEI). An experienced kinesiologist, registered nurse (RN), RD, and licensed practical nurse (LPN) assisted in the study conduction. The details of the program are published elsewhere [10].

### 2.3. Study ethical approval

The UPEI Research Ethics Board (REB) approved the study with the file number 6,008,357, as well as Health PEI REB. The study was performed in accordance with the Helsinki Declaration. A written informed consent was obtained from each participant prior to enrollment in the study. The consent entailed the study objectives and benefits of participation, in addition to complete voluntary participation and the right to withdraw from the study at any time without any drawbacks. Also, confidentiality of the provided information and anonymity of participation were mentioned.

### 2.4. Study instruments

The study employed a self-administered questionnaire that covered participant's sociodemographic characteristics, physical activity, and physical fitness tests: 6-min walk test, chair stand test, shoulder-flex test, sit-and-reach test [13], the one-leg stance test, grip strength test [14], sedentary times [using the Physician Assessment and Clinical Education (PACE) questionnaire] [15], and dietary habits [using the Mediterranean Diet Score (MDS) Questionnaire] [16]. In addition, medical history and anthropometric measurements (weight, height, and waist circumference) were assessed by the RN and LPN. The details of these study instruments are available in a previous study [10].

Physical activity in this study was assessed using the Godin Leisure-Time Exercise Questionnaire [17]. The questionnaire evaluated the number of times, on average, during a typical 7-day period, a participant exercised for more than 15 minutes during free time, undergoing strenuous exercise (e.g., running, jogging, hockey, football, soccer, squash, basketball, vigorous swimming), moderate exercise (e.g., fast walking, baseball, tennis, easy bicycling, volleyball, badminton, and easy swimming), and mild/light exercise (e.g., yoga, archery, fishing from river bank, bowling, and easy walking). The total score was calculated according to Godin (2011) and divided into three categories: active, moderately active, and insufficiently active.

### 2.5. Data analysis

Collected data was analyzed using StataSE 17. Frequencies, means, and standard deviation (SD) were calculated to describe the study sample. Chi-squared test, Pearson correlation test, and Student's t-test were used to evaluate the significant association between two variables.

Binary logistic regression analysis was performed to define the significant predictors (independent variables) for upholding each of the five dependent MetS criteria (outcome variables) after adjusting for the covariates and to estimate the effect of explanatory variables (intervention activities) on each outcome (dependent) variable.

### 2.6. Outcome variables

These analyses included five outcome variables; each of the five MetS criteria was a dependent outcome variable. The measurements at the end of the 12-month study period were considered. The blood pressure measurement was categorized as “High” and given a score of 1 if ≥130/85 mm Hg or if the participant was receiving pharmacotherapy for hypertension; otherwise, blood pressure was categorized as “Normal” and given a score of 0. HbA1c blood level was another dependent outcome variable. If ≥5.7% or if the participant was receiving pharmacotherapy for elevated blood sugar, it was categorized as “High” and given a score of 1; otherwise, it was “Normal” with a score of 0. Triglyceride blood level was the third outcome variable and was given a score of “1” if ≥1.7 mmol/L or if the participant was receiving pharmacotherapy for elevated triglycerides; otherwise, it was given a score of 0. HDL blood level was the fourth outcome variable; high levels according to sex were given a score of 0 and low levels were given a score of 1. Waist circumference measure was the last outcome variable; it was given a score of 1 if it exceeded the recommended measure according to sex; otherwise, it was given a score of 0. The level of significance was considered at *p*-value < 0.05 and confidence interval (*CI*) at 95%.

### 2.7. Covariates

Participants' sociodemographic factors, physical activity (PA) categories, and total scores of physical fitness tests, sedentary time, and MDS were independent variables that were evaluated for association with each of the outcome variables.

## 3. Results

The sociodemographic characteristics of the 278 participants who participated in baseline and 12-month assessments are shown in Table 1. The mean age was 60.5 years, 15.8% were less than 50 years old, and 74.8% of them were females. The majority of participants were married (70.9%), and 27% reported an income of less than $50,000. Moreover, 43.5% of participants reported a bachelor's degree or above. The distribution of participants' characteristics according to the number of their metabolic syndrome criteria is shown in the table. More than half of all participants, regardless of their sociodemographic characteristics, met four or five metabolic syndrome criteria at the baseline of the study.

**Table 1.** Sociodemographic characteristics of the CHANGE study participants, *n* = 278 (percentage is row-wise).

**Characteristics**	**Number of metabolic syndrome criteria**									
	**Total**		**2^a^** ***n* = 34**		**3** ***n* = 68**		**4** ***n* = 105**		**5** ***n* = 71**	

	***n***	**(%)**	***n***	**(%)**	***n***	**(%)**	***n***	**(%)**	***n***	**(%)**
**Age**										
<50	44	(15.8)	10	(22.7)	8	(18.2)	17	(38.6)	9	(20.5)
50–59	68	(24.5)	7	(10.3)	22	(32.4)	19	(27.9)	20	(29.4)
60–69	99	(35.6)	7	(7.1)	24	(24.2)	43	(18.1)	25	(25.3)
70+	67	(24.1)	10	(14.9)	14	(20.9)	26	(38.8)	17	(25.4)
*Mean ± SD*	*60.5 ± 11.7*		*58.4 ± 15.4*		*59.9 ± 11.1*		*61.5 ± 11.4*		*60.6 ± 10.5*	
**Sex**										
Male	70	(25.2)	10	(14.3)	14	(20.0)	29	(41.4)	17	(24.3)
Female	208	(74.8)	24	(11.5)	54	(26.0)	76	(36.5)	54	(26.0)
**Educational attainment**										
≤High school	74	(26.6)	4	(5.4)	18	(24.3)	30	(40.5)	22	(29.7)
Post-sec- < Bachelor's	83	(29.9)	13	(15.7)	19	(22.9)	33	(39.8)	18	(21.7)
Bachelor's degree+	121	(43.5)	17	(14.1)	31	(25.6)	42	(34.7)	31	(25.6)
**Marital status**										
Married	197	(70.9)	26	(13.2)	50	(25.4)	70	(35.5)	51	(25.9)
Unmarried	81	(29.1)	8	(9.9)	18	(22.2)	35	(43.2)	20	(24.7)
**Income**										
<50,000	75	(27.0)	10	(13.3)	18	(24.0)	24	(32.0)	23	(30.7)
50,000–<100,000	123	(44.2)	16	(13.0)	24	(19.5)	56	(45.5)	27	(22.0)
100,000 or more	60	(21.6)	4	(6.7)	23	(38.3)	16	(26.7)	17	(28.3)
Prefer not to say	20	(7.2)	4	(20.0)	3	(15.0)	9	(45.0)	4	(20.0)

Note: ^a^ at risk for metabolic syndrome.

**Figure 1.**
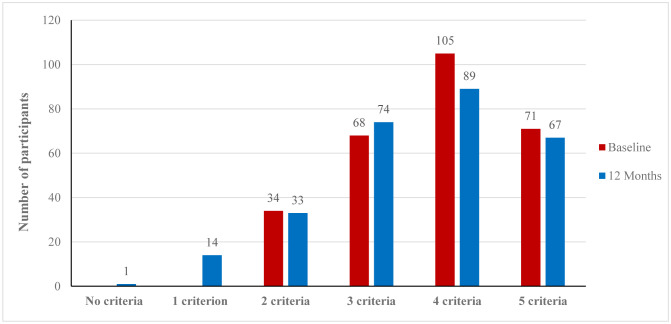
Number of participants with metabolic syndrome criteria at baseline and after 12 months in the CHANGE program study.

Table 2 displays the mean and standard deviation (SD) of the five metabolic syndrome criteria at the baseline and after 12 months of the CHANGE program, along with the prevalence of these criteria according to the participant's number of metabolic syndrome criteria. The table demonstrates that both systolic and diastolic blood pressure means showed a significant decrease at the end of the study compared to the baseline, and the number of participants with high blood pressure (e.g., ≥135/85 mmHg and/or taking hypertension medications) decreased significantly from 232 (83.5%) to 207 (74.5%). Waist circumference presented a significant reduction from 113.4 to 105.3 cm after 12 months, with six participants moving from large to normal waist circumference. The means of HbA1c, triglycerides, and HDL did not show any significant reduction, even though 12 and 11 participants showed a reverse of high HbA1c and low HDL at the end of the study, respectively. Regarding the total number of metabolic syndrome criteria among participants, there was a significant 5% increase in the number of participants with <3 criteria (one without any criteria, and 14 with one criterion), and those with 4 or 5 criteria showed a significant reduction by 5.8% and 1.4%, respectively (Figure 1). The number of MetS criteria at baseline was tested for normality using the Shapiro–Wilk test, and the *p*-value was 0.658, indicating a normal distribution.

**Table 2.** Prevalence of the five metabolic syndrome criteria at baseline and after 12 months among the CHANGE study participants, *n* = 278 (percentage is row-wise).

**Metabolic syndrome criteria**	**Total**		**Number of metabolic syndrome criteria**			
			**<3^a^**	**3**	**4**	**5**

	***Mean* (*SD*)**	***n* (%)**	***n* (%)**	***n* (%)**	***n* (%)**	***n* (%)**
**High blood pressure**					
Baseline	136.7 (17.1)/82.2 (10.6)	232 (83.5)	20 (8.6)	46 (19.8)	95 (41.0)	71 (30.6)
12 months	129.9* (15.1)/78.2* (9.4)	207 (74.5)*	18 (8.7)	48 (23.2)	74 (35.8)	67 (32.4)
**HbA1c ≥ 5.7**						
Baseline	6.27 (1.1)	199 (71.6)	9 (4.5)	32 (16.1)	87 (43.7)	71 (35.7)
12 months	6.25 (1.1)	187 (67.3)*	6 (3.2)	36 (19.3)	78 (41.7)	67 (35.8)
**Triglycerides ≥ 1.7**					
Baseline	1.9 (1.0)	218 (78.4)	7 (3.2)	46 (21.1)	94 (43.1)	71 (32.6)
12 months	1.9 (0.9)	207 (74.5)*	11 (5.3)	45 (21.7)	84 (40.6)	67 (32.4)
**Low HDL**						
Baseline	1.23 (0.3)	125 (45.0)	1 (0.8)	14 (11.2)	39 (31.2)	71 (56.0)
12 months	1.24 (0.3)	125 (45.0)	4 (3.2)	21 (16.8)	33 (26.4)	67 (53.6)
**Large waist circumference**					
Baseline	113.4 (14.8)	273 (98.2)	31 (11.4)	66 (24.2)	105 (38.5)	71 (26.0)
12 months	105.3* (13.7)	267 (96.0)*	41 (15.4)	72 (27.0)	87 (32.6)	67 (25.1)
**Total criteria**						
Baseline	3.8 (1.0)	278 (100.0)	34 (12.2)	68 (24.5)	105 (37.8)	71 (25.5)
12 months	3.6* (1.1)	278 (100.0)	48 (17.3)*	74 (26.6)*	89 (32.0)*	67 (24.1)*

Note: *p ≤ 0.0001, ^a^ at risk for metabolic syndrome (only two criteria at baseline).

Body mass index (BMI) was calculated, and its mean significantly decreased from 35.97 at the baseline to 35.02 at the end of the study (*p* < 0.0001). The prevalence of overweight and obesity at the baseline was 16.2% and 80.9%, respectively, and it was 22.3% and 74.1%, respectively, at the end of the 12 months.

**Figure 2.**
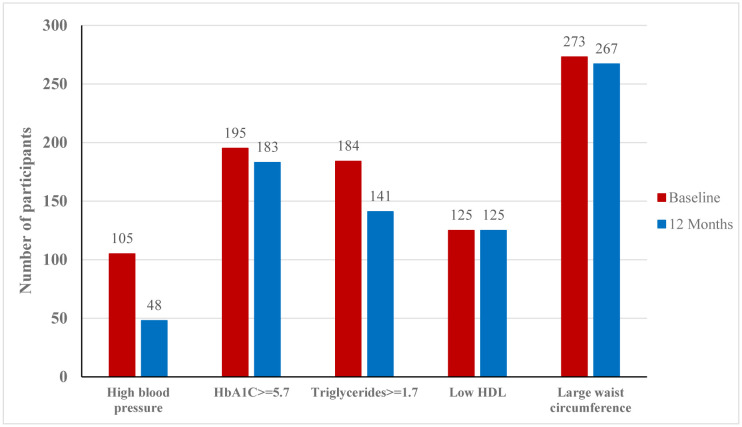
Number of the CHANGE program study participants with each metabolic syndrome criteria after adjusting for medications.

The study found that there was negligible change in the amount of prescribed pharmacotherapy to the participants for high blood pressure, high HbA1c, and triglycerides (as per the definition of MetS) at the end of the study, with roughly 1% reporting reduction in the prescribed dose. However, when focusing solely on the values themselves (after adjusting for taking medication at the baseline and the end of the study), the number of participants with high blood pressure dropped by 54.3%, those with HbA1c dropped by 6.2%, and those with high levels of triglycerides dropped by 23.4%, as illustrated in Figure 2.

The changes in the means and SD of diet, physical activity, and fitness at the baseline and at the end of the 12 months are demonstrated in Table 3, with significant improvements seen for all factors. In addition, the amended performance of participants in the program activities was much better among those who had less than three metabolic syndrome criteria than their counterparts with three or more criteria.

**Table 3.** Means and SD of intervention activities according to the number of metabolic syndrome criteria at baseline and after 12 months among the CHANGE study participants, *n* = 278 (percentage is row-wise).

**CHANGE program intervention activities**	**Total**		**Number of metabolic syndrome criteria**			
			**<3^a^**		**≥3**	

	**Baseline**	**12 months**	**Baseline**	**12 months**	**Baseline**	**12 months**
	***Mean* (*SD*)**	***Mean* (*SD*)**	***Mean* (*SD*)**	***Mean* (*SD*)**	***Mean* (*SD*)**	***Mean* (*SD*)**
**Physical activity**	22.4 (19.0)	38.8 (22.1)*	22.6 (18.2)	44.8 (27.4)*	22.3 (19.2)	37.6 (32.8)*
**MDS**	5.6 (1.8)	8.1 (2.1)*	5.9 (1.7)	8.3 (1.8)*	5.5 (1.7)	8.0 (2.1)*
**Fitness activities**						
6 min walk (m)	482.5 (88.7)	539.0 (96.1)*	503.4 (70.4)	568.9 (122.2)*	478.2 (91.4)	532.9 (98.0)*
Chair stand (#/30 s)	12.0 (2.6)	16.5 (4.2)*	12.3 (2.8)	16.7 (4.7)*	11.9 (2.5)	16.5 (4.2)*
Sit and reach (cm)	0.5 (9.5)	8.1 (8.6)*	−0.09 (7.9)	7.2 (8.9)*	0.6 (9.7)	8.3 (8.6)*
Shoulder flex (cm)	−14.6 (13.2)	−9.3 (10.6)*	−10.2 (9.4)	−3.8 (7.5)*	−15.4 (13.6)	−10.4 (10.8)*
Unipedal open eyes (s)	24.1 (17.1)	33.9 (14.4)*	27.9 (16.3)	37.6 (11.1)*	23.3 (17.1)	33.2 (14.8)*
Unipedal closed eyes (s)	4.2 (6.3)	7.7 (8.5)*	5.5 (7.7)	9.0 (9.5)*	3.9 (5.8)	7.4 (8.3)*
Handgrip (kg)	54.3 (18.9)	65.9 (20.9)*	51.4 (14.7)	63.7 (19.5)*	54.8 (19.6)	66.4 (21.1)*
**Sedentary time/week (hours)**	8.6 (4.2)	7.2 (3.2)*	7.7 (3.6)	6.6 (2.7)*	8.8 (4.3)	7.4 (3.3)*
**Screen time/week (hours)**	3.2 (2.2)	2.6 (1.8)*	2.7 (1.9)	2.0 (1.1)*	3.6 (2.2)	2.7 (1.9)*

Note: *p < 0.0001, ^a^ at risk for metabolic syndrome with two criteria at baseline, but one and no criteria at 12 months

Pearson correlation analysis was performed to examine the intercorrelations between metabolic syndrome criteria and measures of fitness and self-reported physical activity. The analysis revealed that blood pressure was positively correlated with waist circumference (*r* = 0.16, *p* = 0.008); however, it was negatively correlated with HDL (*r* = −0.14, *p* = 0.017), physical activity, 6-min walk test, sit & reach test, unipedal stance test with opened eyes, and grip strength (*r* = −0.12, *p* = 0.04; *r* = −0.18, *p* = 0.003; *r* = −0.12, *p* = 0.04; *r* = −0.2, *p* = 0.001; and *r* = −0.19, *p* = 0.001, respectively). HbA1c was positively correlated with triglycerides and waist circumference (*r* = 0.4, *p* < 0.0001; *r* = 0.24, *p* = 0.0001, respectively) and negatively correlated with physical activity, 6-min walk test, shoulder-flex test, and total MDS score (*r* = −0.18, *p* = 0.024; *r* = −0.14, *p* = 0.005; *r* = −0.35, *p* ≤ 0.0001; *r* = −0.16, *p* = 0.007, respectively). Triglycerides were positively correlated with waist circumference (*r* = 0.13, *p* = 0.034) and negatively correlated with HDL and total MDS score (*r* = −0.35, *p* < 0.0001; *r* = −0.19, *p* = 0.001, respectively). HDL was negatively associated with waist circumference and handgrip strength (*r* = −0.25, *p* = 0.044; *r* = −0.33, *p* < 0.0001, respectively) and positively associated with the shoulder-flex test (*r* = 0.21, *p* = 0.0004). Waist circumference was negatively correlated with 6-min walk, unipedal stance test with open eyes, and shoulder-flex tests (*r* = −0.35, *p* ≤ 0.0001; *r* = −0.19, *p* = 0.002; *r* = −0.36, *p* ≤ 0.0001, respectively) and positively correlated with average daily sedentary hours and screen hours (*r* = 0.24, *p* = 0.0001; *r* = 0.15, *p* = 0.015, respectively).

Table 4 illustrates the binary logistic regression analysis of significant predictors of upholding each metabolic syndrome criterion after adjusting for sociodemographic characteristics and other independent covariates. Participants' age, gender, and educational attainment were significant sociodemographic predictors. Age was a significant predictor for all MetS criteria except for high HbA1c. Relatively older participants (60+ years) were 1.99, 2.3, and 2.6 times more likely to maintain high blood pressure, high triglycerides, and low HDL, respectively, compared to those who aged less than 60 years. On the other hand, older participants (60+) were 98% less likely to preserve large waist circumference compared to their relatively younger counterparts who were <60 years old. Female participants were almost 4 and 31 times more likely to uphold low HDL and large waist circumference, respectively, compared to male participants. However, they were 84% less likely to maintain high triglycerides relative to their male correspondence. Moreover, participants with lower educational attainment were 1.7 times more likely to maintain high levels of HbA1c than their counterparts who reported having a bachelor's degree or above.

**Table 4.** Binary logistic regression of significant predictors of upholding each metabolic syndrome criterion among the CHANGE study participants, *n* = 278.

**Significant predictors**	**Metabolic syndrome criteria**														
	**High blood pressure**			**HbA1c ≥ 5.7**			**Triglycerides ≥ 1.7**			**Low HDL**			**Large waist circumference**		

	***β***	***OR***	**[95% *CI*]**	***β***	***OR***	**[95% *CI*]**	***β***	***OR***	**[95% *CI*]**	***β***	***OR***	**[95% *CI*]**	***β***	***OR***	**[95% *CI*]**
**Sociodemographic characteristics**
**Age**															
<60	0.0	1.0	[RG]				0.0	1.0	[RG]	0.0	1.0	[RG]	0.0	1.0	[RG]
60+	0.69	1.99^a^	[1.13–3.5]				0.83	2.29^b^	[1.28–4.09]	0.96	2.62^b^	[1.44–4.77]	−4.07	0.02^b^	[0.01–0.35]
**Sex**															
Male							0.0	1.0	[RG]	0.0	1.0	[RG]			[RG]
Female							−1.81	0.16^c^	[0.06–0.44]	1.35	3.86^b^	[1.92–7.78]	3.80	31.3^b^	[3.6–27.5]
**Education level**															
<Bachelor's				0.53	1.70^a^	[1.01–2.86]									
≥Bachelor's				0.0	1.0	[RG]									
**CHANGE Program intervention activities**
**Physical activity**															
Active							0.0	1.0	[RG]						
Mod/insufficient							1.07	2.91	[1.37–6.19]						
**Fitness activities**															
6 min walk test													−0.02	0.98^b^	[0.97–0.99]
Shoulder-flex test				−0.10	0.90^c^	[0.87–0.94]	−0.07	0.93^c^	[0.90–0.97]	−0.05	0.95^b^	[0.93–0.98]	−0.11	0.90^b^	[0.83–0.97]
Sit-reach test				−0.05	0.95^a^	[0.88–0.96]									
Unipedal stance test (opened eyes)	−0.04	0.97^b^	[0.94–0.99]												
Unipedal stance test (closed eyes)													−0.16	0.86^b^	[0.77–0.95]
Handgrip										−0.03	0.97^a^	[0.94–0.97]	−0.18	0.95^a^	[0.86–0.93]
**Average screen time**							0.22	1.24^a^	[1.01–1.53]					

Note: ^a^ *p* < 0.05, ^b^ *p* < 0.01, ^c^ *p* < 0.0001.

Regarding diet, physical activity, and fitness, physical inactivity was a significant predictor for maintaining high triglycerides. Moderately or insufficiently active participants were almost three times more likely to maintain high triglycerides compared to physically active ones. For the fitness activities, the study revealed that each meter increase in the 6-min walk test was associated with a 2% decrease in waist circumference. Further, each centimeter increase in the shoulder-flexibility test was associated with a reduction of 10% in HbA1c, 7% in triglycerides, and 10% in waist circumference; however, it was related to a 5% improvement in HDL blood levels (inverse association low *vs*. high). Also, each unit improvement in the sit-reach test was connected with a 5% significant reduction in HbA1c blood level. Further, each unit increase in the balance-with-eyes-open test was associated with a 3% reduction in blood pressure, while the same increase in balance with eyes closed was associated with a 14% reduction in waist circumference. Each kilogram increase in grip strength was associated with a 3% increase in HDL blood level and a 5% reduction in waist circumference. Finally, average daily screen time was a significant predictor of high triglyceride blood levels. Each increase in one hour in daily screen time was associated with a 24% increase in triglyceride blood levels.

## 4. Discussion

The purpose of this analysis was to examine the impact of the CHANGE program on the reversal of one or more metabolic syndrome criteria among community members and identify significant predictors of upholding individual metabolic syndrome criteria in a period from 2020 to 2023. Our findings suggest that a one-year community-based diet and exercise program leads to significant reductions in blood pressure and waist circumference and the mean number of metabolic syndrome components. Further, more than 5% of individuals with MetS at baseline no longer had MetS at follow-up, as they reduced their mean values for risk factors and were no longer taking medication for those risk factors. Taken together, these findings suggest that reversing one or more MetS criteria can be achieved in a community setting by adopting a healthy lifestyle in terms of regular physical activity and improved diet.

### 4.1. Number of MetS reversed criteria and estimated financial impact of the CHANGE program

The number of reversed MetS criteria is the most critical result of this study. Following the CHANGE program for 12 months was sufficient to reduce blood pressure (BP) to the point where medicine was no longer needed for 25 participants. Further analysis showed that up to 10.57% of changes in systolic blood pressure could be significantly explained by weight changes. This interesting finding provides an optimistic vision for the possibility of the reduction of cardiovascular diseases (CVD), which is indicated by the Global Burden of Disease study as the worldwide leading cause of death with 17.8 million related deaths in 2017 [18]. Moreover, this is a very impactful result, knowing that almost 20% of Canadians have hypertension [19],[20] and the average annual healthcare cost of a hypertensive patient is $7985 in 2024 dollars [21]; accordingly, the CHANGE program could save $199,000 among the study participants due to reductions in blood pressure alone, which represents almost half the budget of the program over its first 3 years. Although beyond the scope of the present study, a more in-depth cost-benefit analysis is warranted in the future.

Moreover, by the end of the study, 12 participants had normal levels of HbA1c; they were also no longer taking glucose-lowering medications. Protecting people from developing type 2 diabetes mellitus (and its enormous and serious complications) by just adopting this simple program is a noticeable outcome. Moreover, the Canadian government reported that 9.4% of Canadians (3.7 million people) have diabetes; their full healthcare costs [direct healthcare costs and unnecessary costs (indirect) such as low productivity] were estimated to be $27 billion in 2018 [22]. Further, the out-of-pocket annual expenses for a person with type 2 diabetes could reach $10,014, as reported in the 2022 report of Diabetes Canada [23]. Therefore, the CHANGE program could simply save approximately $120,000 out-of-pocket costs in our study.

Additionally, the CHANGE program succeeded in reversing high triglycerides in 11 participants and large waist circumference in 6 participants, and 17 participants decreased their BMI from obese to overweight. These findings could be added to the cost-effectiveness of this program. This vital result is supported by Anis' study (2010), who reported that in 2006, $6.0 billion were the total direct costs attributable to overweight and obesity in Canada, where obesity is responsible for 66% of the costs [24].

Furthermore, the study revealed that one participant became free from any MetS criteria and 14 individuals had only one criterion by the end of the study. Although these 15 participants were among those who were at risk of MetS (had only two criteria at enrollment), the study could protect them and prevent the development of MetS; these individuals became able to enjoy their life with minimal/no medications and were more productive, adding to the huge reduction in their healthcare costs.

These findings suggest that this type of community-based program may lead to substantial cost savings for both individuals and the healthcare system, highlighting the need for more in-depth cost-benefit analyses in the future.

Additionally, the study results revealed that participants with less than three MetS criteria performed better in the program activities than those with three or more criteria. This could be explained as the former were likely to be in a better initial physical condition, making it easier to engage in and sustain physical activity; their eventually better metabolic health could lead to higher energy levels and better recovery from physical exertion. Moreover, they could feel more confident and motivated to participate in fitness programs, compared to those with three or more MetS criteria. These might be less motivated to engage in healthy behaviors due to physical limitations, such as reduced mobility, endurance, and strength, hindering their ability to participate fully in physical activities; also, these might have more ingrained unhealthy habits that are harder to change. Some strategies could enhance the performance of participants with more MetS criteria, for example, tailored fitness programs aiming for gradual progression and employing low-impact activities such as walking and cycling, which are easier on the joints and more accessible for those with physical limitations. The same applies to personalized diet plans that consider the special needs and preferences of these participants. Moreover, behavioral support and motivation can help these participants in terms of encouraging small progress to build confidence and motivation, particularly withing support groups. Further, it is important to consider mental health support and stress management programs to address any psychological barriers.

### 4.2. Sociodemographic factors and reversal of MetS criteria

This intervention study pointed out that age was an important significant independent predictor that should be considered when targeting improving MetS criteria; the older the person (60+), the more likely they are prone to maintain three MetS criteria (high blood pressure and triglycerides and low HDL) compared to their younger counterparts. These findings are well-established in several evidence-based preceding studies; for instance, Holzer et al. (2013) reported that age is associated with significant changes in the composition of HDL, which alters its function and may contribute to developing CVD [25]. Moreover, Morvaridzadeh et al. (2024) [18] reported that an increased risk of heart attack and stroke and an amplified risk of mortality [26],[27] are significantly associated with low HDL levels, which further decrease with aging. Furthermore, HDL is functionally impaired in the elderly by several chronic diseases such as diabetes mellitus, kidney diseases, and metabolic syndrome, which draw attention to the substantial role of aging in determining HDL function and, subsequently, CVD [28]–[31]. Earlier studies, including a meta-analysis of 23 studies conducted in Asia, emphasized the association between low levels of HDL and the increased risk of stroke, cardiac attack, and coronary artery disease [18],[32]. Although age is found to be a prominent predictor of most MetS criteria, great attention should be drawn to the 44 persons who were younger than 50 years old and were included in the study; nine of them had all the five criteria of MetS.

Triglycerides are the most common type of fat that is stored in a person's diet. Increased levels of triglycerides increase arterial atherosclerosis and subsequently high blood pressure, CVD, and stroke. Aging is associated with noticeable alteration in lipid digestion, absorption, and metabolism, with increased blood levels that will aid in an augmented risk of serious chronic diseases [33]. This is an alarming finding that highlights a crucial call for healthcare professionals and public health policy planners regarding the vital need of health promotion campaigns about the importance of adopting a healthy lifestyle in terms of being physically active early in life and among this age group. In addition, controlling other reversible factors, such as high blood pressure, triglycerides, and waist circumference, requires changing to a healthy diet, which will help in controlling blood lipids and facilitate the reversal of MetS criteria as early as possible before confronting the irreversible factor of aging.

On the other hand, the study revealed that older participants were less likely to maintain a large waist circumference than younger participants. This was an anticipated finding, as elderly people are expected to have unintentional weight loss and consequently smaller waist circumference. This change is more common in men at an earlier age than women. Physiological changes such as suppressed appetite and lower release of anabolic hormones, chronic physical health conditions that can have a catabolic effect (e.g., diabetes mellitus) or be associated with loss of appetite due to disease pathophysiology or pharmacologic side effects, and psychological and social changes are also factors related with losing weight among frail people [34],[35].

Sex was another significant predictor for upholding some MetS criteria. Female participants were more likely to preserve large waist circumference and low HDL relative to male participants. Large waist circumference, particularly when it reflects visceral fat accumulation, is associated with increased health risks such as cardiovascular disease, diabetes, asthma, and metabolic syndrome; it is used as a marker for central obesity and has a substantial role in predicting health outcomes [36],[37]. Hormonal differences between sexes can influence fat distribution. Testosterone in men and estrogen in women affect where fat is stored in the body. Men tend to accumulate more visceral fat around the abdomen, while women often have more subcutaneous fat around the hips and thighs. The former is more metabolically active, impacting HDL levels differently. However, these effects deteriorate with age; this mechanism starts much earlier in men than in women, which could justify the preservation of large waist circumference among women compared to men [34]. Moreover, excessive gestational weight gain could be related to failure to lose the extra weight gained and preservation of a large waist circumference later in life [38]. Further, metabolic differences could be another reason. Women typically have a lower basal metabolic rate compared to men, which can contribute to easier weight gain and difficulty losing abdominal fat. Regarding sex differences in serum lipids, the study found that females were more likely to maintain low HDL blood levels than males. The correlation analysis emphasized this finding, as HDL showed a negative correlation with waist circumference, and female participants had larger waist circumference than males. This finding is in agreement with previous studies based on physiological changes taking place among aging women. Estrogen hormone rises HDL cholesterol levels and enhances clearance of LDL cholesterol by the liver. Therefore, it is expected to find a reduction in HDL/LDL ratio among postmenopausal women, which increases their risk for CVD [39]–[42]. On the other hand, females in the present study were less likely to uphold higher levels of triglycerides compared to males. This result is in agreement with a retrospective study among over 230,000 individuals from 2009 to 2015, where triglycerides were lower in older women than men [43].

There are different ways to address these sex differences, for example, through tailored dietary and exercise programs that focus on reducing abdominal fat and increasing HDL. Health education could be another way to increase women's awareness about the importance of maintaining a healthy waist circumference and HDL levels, the benefits of physical activity, and how to incorporate these into daily routines. Moreover, promoting regular health check-ups for women, including measurements of waist circumference and lipid profiles, creating supportive environments such as safe spaces for physical activity, access to healthy foods, and workplace wellness programs, advocating for policies that promote gender equity in health, and addressing mental health issues that may impact women's ability to manage their weight and lipid levels are all vibrant approaches that can help mitigate these sex differences and promote better health outcomes for women.

Educational attainment was found to be a significant predictor for keeping high levels of HbA1c. This was an expected finding; individuals with higher educational attainment will be more understanding of the risk of uncontrolled HbA1c blood levels, which in turn will contribute to severe diabetes complications such as CVD, stroke, chronic renal disease, vision impairment, and nerve damage. This result concurs with well-established previous findings that demonstrated that people with lower educational levels are more likely to have more prevalent diabetes [44],[45]. Moreover, it has been reported that well-educated people are more careful regarding their nutritional behaviors, compliance with medications, and routine checking for blood glucose levels, and have generally better control of the disease [46]. This is an interesting finding, shedding light on the importance of providing regular educational sessions in community health centers or primary healthcare settings for less educated people regarding the serious complications of having high HbA1c levels and the importance of adopting a healthy lifestyle to prevent the development of diabetes mellitus or to delay the onset of its complications.

Additionally, it is well-established that MetS is more prevalent in rural areas than urban residences [47],[48]. Therefore, healthcare workers in PEI, as a rural area, ought to engage in the prevention and promotion of healthy lifestyles through education and guidance to help reduce the prevalence of MetS among the general community and, particularly, those at higher risk.

### 4.3. Physical activity

The study revealed that physical activity was a significant negative predictor for possessing unfavorable triglyceride blood levels. Triglycerides are unlike other blood lipids/cholesterol in their ability to produce energy. This is particularly true during aerobic exercises, which are the most effective method to decrease triglycerides; the harder the exercise, the higher the acute lowering of blood triglycerides post-exercise. This is supported by earlier studies that explained the decrease in blood triglycerides following physical activity because of increased activity of lipoprotein lipase (LPL)-mediated hydrolysis of circulating lipoprotein particles, increasing availability of fatty acids necessary for adipocyte uptake. Moreover, in endurance-trained athletes, their low body fat contents contribute, through the same mechanism, to lower triglyceride blood concentrations [49],[50].

In contrast to physical activity, daily screen time was a significant independent positive predictor of high triglyceride blood levels after adjusting for sociodemographic factors and other covariates. This important finding reflects the recent attention to the health impact of engaging in sedentary behaviors. Several studies have discussed the role of physical activity on health and blood lipids; however, little is known about the role of sedentary behaviors. The findings of this study are in agreement with a preceding study that reported a positive association between screen time and triglycerides among only normal-weight people; in contrast, our study found the same association among overweight and obese participants [51]. Another study reported that prolonged sedentary time is associated with an increased risk of obesity, diabetes, cardiovascular disease, and all-cause mortality [52].

Defining the significant positive and negative predictors of triglycerides is of great importance, together with the positive correlation between triglycerides and HbA1c and waist circumference and the negative correlation with HDL found here. Therefore, simply adopting physical exercise (moderate to vigorous) will assist in improving the other three MetS criteria, which will indirectly improve high blood pressure. This highlights how one simple, cost-free intervention can help in reversing one or more MetS criteria.

### 4.4. Physical fitness

The study revealed that lack of physical fitness was an independent significant predictor for upholding any of the five MetS criteria. At least one of the physical fitness activities was inversely associated with the presence of each MetS criteria. For instance, handgrip, as a simple index of muscle strength, was inversely associated with large waist circumference and high HDL. This finding supports the results of previous epidemiologic studies conducted in different countries such as China [53],[54] and Saudi Arabia [55]. These studies concluded that handgrip strength is independently and inversely related to increased abdominal obesity and confirmed its association with the incidence of MetS. Handgrip relates to muscle mass and higher basal metabolic rate, leading to a reduction in central obesity; it improves glucose uptake by muscles, reducing elevated blood glucose and, consequently, HbA1c. In addition, it enhances fat utilization and decreases high triglycerides. Moreover, the test is associated with higher HDL levels due to increased muscle mass.

The study also found that poor balance was a significant independent contributor of having high blood pressure and vice versa. This is an imperative finding since hypertension is a major global public health concern based on its substantial contribution to cognitive dysfunction, CVD, and all-cause mortality [56],[57]. Therefore, enhancing balance, particularly among elderly people, will be associated with a significant reduction in blood pressure and the risk of serious illnesses/falls. This finding is supported by a preceding study, which concluded that hypertensive people showed lower balance compared to normotensive ones [58]. Better balance is often correlated with overall physical fitness, which indirectly helps manage blood pressure through regular physical activity. Moreover, improving balance among the elderly will significantly reduce falls, the leading cause of morbidity and mortality-related injuries among old people, mainly due to impaired balance [59]. This fall reduction will likely be associated with significant savings, knowing that the direct cost of fall-related injuries in elder Canadians aged 65 years or older was estimated to be $5.6 billion in 2018 [60].

Additionally, shoulder flexibility was found to be a significant inverse contributor to almost all MetS criteria except for high blood pressure. This was an interesting finding; by enhancing shoulder flexibility, which protects against injury, many other serious health problems such as type 2 diabetes mellitus, lipidemia, and obesity could also be improved. This might be explained by better shoulder flexibility occurring among physically active people who enjoy normal body weight. This result paralleled a previous study that found that more limited shoulder mobility is found in people with diabetes than in the control group [61]. This limitation can increase the risk of impaired daily functions that require flexible upper extremities [62]. Type 2 diabetes is known to be associated with limited functions of smaller joints in the hands and feet, but a limitation of big joints is less documented. However, diabetes-related metabolic abnormalities, which affect periarticular and skeletal connective tissues, could be the underlying reason for this limited shoulder flexibility [63]–[65]. Further research, to better understand the causes of diabetic limited shoulder flexibility, is recommended to prevent additional harmful limitations in movement and function. Improved shoulder flexibility indicates improvements in general physical activity, which helps reduce fat and is associated with an active lifestyle, improving HDL levels.

In addition, other employed fitness activities have a great impact on MetS. For example, a 6-min walk helps in reducing central obesity by burning calories; it improves cardiovascular health and reduces vascular resistance, leading to decreased high blood pressure; it also enhances insulin sensitivity and lowers fasting glucose, which assists in lowering HbA1c. It also improves lipid metabolism and increases HDL production. Regarding the sit-and-reach test, it indicates better flexibility, which is associated with a more active lifestyle that helps manage blood sugar levels and HbA1c blood levels. The chair-stand test increases muscle mass, boosts metabolism, and improves central obesity; it also improves endothelial function and reduces arterial stiffness, which help in reducing high blood pressure. Moreover, the test promotes the utilization of triglycerides, which in turn helps in the reduction of high triglycerides.

### 4.5. Pharmacotherapy impact

The study results reported a negligible change in the amount of prescribed pharmacotherapy for high blood pressure, high HbA1c, and triglycerides by the study end, despite significant improvements in health markers. The negligible change could be attributed to several factors. For instance, the adherence to medical guidelines by physicians who may be cautious about altering medication regimens, preferring to observe sustained improvement in health markers over a longer period before making changes to prescribed pharmacotherapy. This approach helps physicians to prevent relapses and complication risks and to ensure patient safety. Moreover, considering individual variability in response to the CHANGE program may require some patients to continue their medication regimen; alternatively, severe baseline conditions may necessitate ongoing pharmacotherapy despite improvements. In addition, the study duration may have been sufficient to show improvements in health markers but not long enough to warrant a reduction in medication, which might be necessary to achieve optimal control. Furthermore, the correlation of prescribed pharmacotherapy with significant improvements in health markers could be explained as the CHANGE program activities enhanced the effectiveness of medications resulting in a better control of blood pressure, HbA1c, and triglycerides, or by a synergistic effect of combined pharmacotherapy and the program activities leading to significant health improvements.

### 4.6. Mediterranean diet score

Although the study found that MDS was not a significant predictor for maintaining MetS criteria, it might have an indirect contribution as it was negatively correlated with HbA1c and triglycerides blood levels. Adopting a Mediterranean diet is significantly and substantially associated with lower levels of blood glucose and lipids. Wang et al. (2022) reported that high blood glucose was positively related to the consumption of modern dietary patterns (excess intake of red meat) and negatively related to fruit-milk dietary patterns [66]. In addition, Luna-Castillo et al. (2022) attested to the importance of the Mediterranean diet as the most efficacious diet in lowering blood triglycerides [67]. They added that diet interventions to modify the pool of macronutrient caloric restriction are necessary to reduce blood triglyceride levels. This could mean that the significant improvement in MDS score at the end of the study might be indirectly associated with the lower number of participants with high HbA1c and triglyceride levels.

## 5. Study strengths and limitations

Although the study showed very interesting and significant results, some strengths and limitations should be highlighted. The program included participants from seven sites across PEI, but the distance for some individuals living in rural settings may still have been a barrier to participation. The study was based in a primary care setting and was performed in a largely rural province with limited access to healthcare services relative to other Canadian provinces. This increases the generalizability of our findings to other rural, underserved populations. However, our study did not include a control group, nor were participants randomly selected to participate. In addition, the first cohort was conducted during the pandemic, and some program sessions were conducted virtually, which may affect the performance levels of participants. Finally, another potential limitation of the study stems from considering the eligibility criterion of having at least two MetS criteria, which inherently limited the possible directions of change in participants' number of MetS criteria during follow-up. The potential for participants to reduce to zero or one MetS criteria at follow-up reflects the success of the intervention. However, participants cannot move upward from lower groups (since none were enrolled with fewer than two criteria), and no participants can exceed five criteria (the ceiling of the MetS criteria) if they present a deterioration in their health statuses. This might create a directional skew in the interpretation of changes in MetS criteria (i.e., improvements are possible across the spectrum, but worsening can only occur up to a threshold of five criteria). Future studies could consider enrolling participants across a wider range of baseline MetS criteria to provide a more balanced view of both potential improvements and deteriorations.

## 6. Conclusions

Metabolic syndrome criteria could be reversed by adopting a healthy lifestyle in terms of regular physical activity, physical fitness exercises, and following a Mediterranean diet. This healthy lifestyle should be implemented early in life to promote health and prevent the development of chronic conditions such as hypertension and diabetes mellitus and their subsequent serious complications. MetS criteria are intercorrelated; therefore, reversing one or more of them could help in improving and/or reversing the others. Our results highlight the benefits of a relatively low-cost, 1-year lifestyle program delivered by a kinesiologist and registered dietitian. Healthy lifestyle changes should be considered a line of treatment parallel to pharmacological solutions and be introduced to people with MetS through their healthcare professionals. Further research is recommended to examine gender differences in the impact of the CHANGE program among different age groups and to explore the impact of these types of programs on direct and indirect healthcare costs for participants in diverse communities.

## Use of AI tools declaration

The authors declare they have not used Artificial Intelligence (AI) tools in the creation of this article.
